# Supplementary material for: “I am afraid of being treated badly if I show it”: A cross-sectional study of healthcare accessibility and Autism Health Passports among UK Autistic adults
Source: PLoS One. 2024 May 29;19(5):e0303873. doi: 10.1371/journal.pone.0303873 (PMC11135756; doi:10.1371/journal.pone.0303873)
Supplement: S1 Appendix — (DOCX) [file pone.0303873.s001.docx]

# AHP Survey v1 28.1.22

# Consent page

|  | **Tick Box** |
| --- | --- |
| 1. I have read and understood the information sheet for this study. |  |
| 1. My questions about the study have been answered, and I understand that I am free to ask Aimee questions at any time during this study. |  |
| 1. I know that I can change my mind about taking part in the study, and delete my responses by closing my browser. |  |
| 1. I consent to taking part. |  |
| 1. I am happy for the information I provide to be used (anonymously) in academic papers, other formal research outputs (journals and conferences) and on social media. |  |
| 1. I agree to the researchers processing my personal data in accordance with the aims of the study described in the Participant Information Sheet. |  |

If you agree with all statements listed above, click **YES** to be taken to the survey.

If you disagree with any of the statements above, click **NO** and you will leave this page.

This study is being conducted by Swansea University, School of Health and Social Care.

**Thank you for your participation in this study. Your help is very much appreciated.**

# Part 1: About You

**There are four Parts in the survey. This first part contains 13 questions. It should take around 5 minutes to complete this section**

Q1: Are you autistic?

*This includes people who have been given a diagnosis of older labels including Asperger’s Syndrome, High Functioning Autism or PDD NOS.*

- Yes
- No

[**Skip: if answer No, directed out of the survey]**

Q2: Have you been formally-diagnosed or do you self-identify as autistic?

*Please Note: we fully acknowledge the validity of self-diagnosis. This information is asked because it is requested by journals, where we hope to publish our research.*

- Self-identify
- Undergoing diagnosis
- Formally diagnosed
- Other, please specify [open text box]

Q3: What is your **preferred** communication method?

*Please choose one answer*

- Speaking
- Sign language
- AAC (Alternative and augmentive communication, including apps such as Coughdrop)
- Other, please specify [open text box]
- Prefer not to say

Q4: Do you sometimes use different communication methods, for example in times of stress Please describe which other methods you use and when.

*You can give as much or as little detail as you like. For example: Aimee from the research team would say: I use messenger apps to write what I want to communicate to save energy when I am very tired.*

[open text box]

Q5: Do you ‘mask’ your Autistic behaviour?

*Masking is a term used to describe when Autistic people change their behaviour, such as reducing stimming, to make it more accepted by neurotypical people. It is also sometimes known as camouflaging.*

- Always
- Mostly
- Sometimes
- Rarely
- Never
- Prefer not to say

Q6: What is your gender identity?

- Woman (also known as cis-gendered woman)
- Intersex
- Man (also known as a trans man)
- Non-binary
- Other, please specify [open text box]
- Prefer not to say

Q7: What is your age?

[open text box]

Q8: What is your race/ethnicity?

*Please Note - these have been chosen according to the census - if None of these are the words you would use to describe your race or ethnicity please choose other and type the right word into the box*

**White**

- English, Welsh, Scottish, Northern Irish or British
- Irish
- Gypsy or Irish Traveller
- Any other White background

**Mixed or Multiple ethnic groups**

- White and Black Caribbean
- White and Black African
- White and Asian
- Any other Mixed or Multiple ethnic background

**Asian or Asian British**

- Indian
- Pakistani
- Bangladeshi
- Chinese
- Any other Asian background

**Black, African, Caribbean or Black British**

- African
- Caribbean
- Any other Black, African or Caribbean background

**Other ethnic group(s)**

- Arab
- Any other ethnic group
- Other, please specify [open text box]
- Prefer not to say

Q9: Do you have any physical/mental health conditions or disabilities lasting or expected to last 12 months or more?

*Please* include things **other** than being Autistic here

- Yes
- No
- Prefer not to say

**[No and Prefer not to say skip to Q12]**

# Q10: If Yes: Do any of your conditions or illnesses reduce your ability to carry out day-to-day activities?

- Yes, a lot
- Yes, a little
- Not at all
- Prefer not to say

Q11: Do you feel like you currently receive enough support for your autism and any co-occurring conditions in your day-to-day life?

- Strongly agree
- Agree
- Neither agree or disagree
- Disagree
- Strongly disagree
- Prefer not to say

Q12: What is your highest completed level of education?

None

GCSEs / Standard Grades / other equivalent high school qualifications

A Levels / Highers / other equivalent high school qualifications

National Vocational Qualification or equivalent

Undergraduate degree

Taught postgraduate degree (Masters or equivalent)

PhD

Other (please specify) [open text box]

Prefer not to say

Q13: Where do you currently live in the UK?

- England
- Scotland
- Wales
- Northern Ireland
- Other, please specify [open text box]
- I do Not live in the UK

**[I do Not live in the UK redirects people out of the survey]**

# Part 2: About you and health care

**In this Part, you will be asked some questions about your experiences with healthcare and how it interacts with your communication, sensory and pain experiences. There are 17 questions. It should take around 5-10 minutes to complete this section**

For each statement please choose **one** option to complete the sentence. If you feel like adding more detail about any of these topics, a box is provided at the end of this Part.

**[NB: to be presented in a matrix in Qualtrics]**

Q1: When I experience pain, injury, or discomfort I am ______aware of it

- Always
- Mostly
- Sometimes
- Rarely
- Never

Prefer not to say

Q2: When I experience recurrent symptoms or problems that may be intermittent, such as occasional pain, my decision and efforts to seek diagnostic healthcare appointments are ______ delayed

- Always
- Mostly
- Sometimes
- Rarely
- Never
- Prefer not to say

Q3: When I have to telephone a healthcare services, for example to book an appointment, I ______ feel anxious

- Always
- Mostly
- Sometimes
- Rarely
- Never
- Prefer not to say

Q4: When I have to telephone a healthcare services, for example to book an appointment, I ______ delay making the telephone call

- Always
- Mostly
- Sometimes
- Rarely
- Never
- Prefer not to say

Q5: Sensory experiences in healthcare service waiting rooms ______ make me anxious

- Always
- Mostly
- Sometimes
- Rarely
- Never
- Prefer not to say

Q6: The presence of other patients in healthcare service waiting rooms ______ makes me anxious

- Always
- Mostly
- Sometimes
- Rarely
- Never
- Prefer not to say

Q7: When communicating with healthcare professionals using my preferred type of communication (eg: speaking, sign language, AAC), I ______ experience frustration or misunderstandings

- Always
- Mostly
- Sometimes
- Rarely
- Never
- Prefer not to say

Q8: Healthcare appointments ______ make me anxious

- Always
- Mostly
- Sometimes
- Rarely
- Never
- Prefer not to say

Q9: In healthcare appointments I ______ mask my Autistic communication style or behaviours

- Always
- Mostly
- Sometimes
- Rarely
- Never
- Prefer not to say

Q10: When I am asked to describe physical symptoms I am ______ confident I will be understood

- Always
- Mostly
- Sometimes
- Rarely
- Never
- Prefer not to say

Q11: When I am asked to describe pain I ______ have difficulty

- Always
- Mostly
- Sometimes
- Rarely
- Never
- Prefer not to say

Q12: When healthcare professionals ask me detailed questions or give me lengthy verbal instructions in a consultation I ______ find it difficult to understand

- Always
- Mostly
- Sometimes
- Rarely
- Never
- Prefer not to say

Q13: When I am emotional or distressed in a healthcare consultation or setting, my communication skills are ______ reduced

- Always
- Mostly
- Sometimes
- Rarely
- Never
- Prefer not to say

Q14: Sensory experiences within health care appointments are ______ difficult for me.

- Always
- Mostly
- Sometimes
- Rarely
- Never
- Prefer not to say

Q15: I find that lengthy instructions, such as when to fill prescriptions, have tests, and make follow-up appointments, are ______ easy to understand

- Always
- Mostly
- Sometimes
- Rarely
- Never
- Prefer not to say

Q16: With regard to adhering to post-appointment instructions (such as wound care or medications) I manage to ______ follow instructions exactly

- Always
- Mostly
- Sometimes
- Rarely
- Never
- Prefer not to say

Q17: Is there anything you would like to tell us about your healthcare experiences excluding maternity services (we will ask you about that in Part 5).

*You can write as much or as little as you like*

*You could focus on:*

- ***Communication*** *with health professionals*
- ***Sensory*** *issues whilst seeking healthcare*
- *Any particularly* ***good or bad care*** *that you have received*
- *How that made you feel.*

*If it helps, think about your last three healthcare appointments.*

[open text box]

# Part 3: About you and Autism Health Passports

**In this Part, we will ask you about Autism Health Passports. There are 14 questions. It should take around 5-10 minutes to complete this section**

*Autism health passports are physical or electronic documents for autistic people to complete in advance of treatment, with the idea that they may help to communicate needs (such as sensory or communication needs) in healthcare settings.*

*One of the most well-known examples of this in the UK is the ‘My Hospital Passport’, which you can see below:*

**

Q1: How much do you know about Autism Health Passports?

- I know a lot about them
- I know a little bit about them
- I had Not heard of them before this survey
- Prefer not to say

Q2: Have you ever seen an Autism Health Passport?

- Yes
- No
- Unsure
- Prefer not to say

Q3: Do you use an Autism Health Passport with health professionals?

- Always
- Mostly
- Sometimes
- Rarely
- Never
- Prefer not to say

Q4: If you have ever tried to use an Autism Health Passport with a health professional, can you please tell us about your experiences.

*You can write as much or as little as you like.*

*It would be helpful if you could say what kind of health professional (eg: doctor, physiotherapist) you are talking about.*

[open text box]

**The next set of questions will ask you how much you agree or disagree with some statements about how Autism Health Passports could impact health care.**

Q5: I feel that a completed Autism Health Passport would be, helpful in communicating enough information about my specific autistic presentation.

Strongly agree

- Agree
- Neither agree or disagree
- Disagree
- Strongly disagree
- Prefer not to say

Q6: I feel that a completed Autism Health Passport would have the ability to adequately inform medical professionals about my co-occurring conditions (e.g. epilepsy, EDS).

- Strongly agree
- Agree
- Neither agree or disagree
- Disagree
- Strongly disagree

Q7: I feel that a completed Autism Health Passport would accurately convey my overall health and well-being.

- Strongly agree
- Agree
- Neither agree or disagree
- Disagree
- Strongly disagree
- Prefer not to say

Q8: I feel that a completed Autism Health Passport would reduce the need for me to give the same information to different members of staff? (*E.g. GP to midwife, midwife to Health Visitor*).

- Strongly agree
- Agree
- Neither agree or disagree
- Disagree
- Strongly disagree
- Prefer not to say

Q9: I think a completed Autism Health Passport would help me receive the same care quality as a non-Autistic person.

- Strongly agree
- Agree
- Neither agree or disagree
- Disagree
- Strongly disagree
- Prefer not to say

Q10: I feel that a completed Autism Health Passport would be useful in routine appointments.

- Strong agree
- Agree
- Neither agree or disagree
- Disagree
- Strongly disagree
- Prefer not to say

Q11: I feel that a completed Autism Health Passport would be useful in emergency medical situations.

- Strong agree
- Agree
- Neither agree or disagree
- Disagree
- Strongly disagree
- Prefer not to say

Q12: I feel that a completed Autism Health Passport would be useful to effectively communicate my needs in-between appointments *eg: when booking appointments).*

- Strong agree
- Agree
- Neither agree or disagree
- Disagree
- Strongly disagree
- Prefer not to say

**The last two questions in this Part ask you to tell us about your thoughts on Autism Health Passports in your own words.**

***You can write as much or as little as you like.***

Q13: What barriers do you think there are to using Autism Health Passports in healthcare settings?

*You can think about the perspective of yourself, Autistic people generally or health professionals here.*

[open text box]

Q14: Would you like to tell us anything else you think about Autism Health Passports or any aids that you use to try to improve communication in healthcare settings?

*You can think about any tools or summaries of information that you have created yourself as well as ones that you have been given or found online*

[open text box]

# Part 4: About your maternity care experiences

In this section we are interested in finding out about your maternity care experiences. The questions are organised into six sections on pregnancy, pregnancy loss, antenatal care, birth, infant feeding and postnatal care. You will not see all of the questions, depending on your experiences. This section will take between 5-30 minutes, depending on your experiences and how much you tell us.

***You are welcome to skip any questions.***

## Pregnancy and maternity care

In this section, we ask you questions about sensory issues during pregnancy and your experiences of maternity care. There are 13 questions in this section.

**Thinking about sensory issues…**

Q1: When you were pregnant, did you experience increased sensory issues?

*Please tick one option for each category*

**[matrix table – options: always to never 5 point scale]**

- Light
- Sound
- Touch
- Smell
- Other, please specify [open text box]
- I did not experience increased sensory issues

**[I did not experiences increased sensory issues skips Q2]**

Q2: Did the increased sensory challenges of pregnancy lead to increased:

*Please tick one option for each category*

**[matrix table – options: always to never 5 point scale]**

- Discomfort
- Distress
- Meltdowns/shutdowns
- Anxiety
- Other, please specify [open text box]

**Thinking about attending appointments, and receiving care during procedures or birth…**

Q3: Did you have somebody who attended your appointments with you?

*By somebody, we mean a partner, family member, friend, doula or similar,* ***not*** *a health professional*

- Always
- Mostly
- Sometimes
- Rarely
- Never
- *Prefer not to say*

***[people who say ‘always’ skip to Q9]***

*Q4: If you did not always have somebody attend with you, was this because of restrictions around the COVID-19 pandemic?*

- *Yes*
- *No*
- *Don’t know*

***[Yes answer only directed to Q5; others go to Q6]***

*Q5: If Yes, would you have wanted to have somebody to come with you?*

- Always
- Mostly
- Sometimes
- Rarely
- Never
- *Prefer not to say*

Q6. If somebody attended with you, who were they?

*Please tick all that apply*

- Partner
- Friend or family member
- Doula
- Other (please specify)
- Prefer not to say

Q7. If somebody attended with you, did they communicate on your behalf?

- Always
- Mostly
- Sometimes
- Rarely
- Never
- Prefer not to say

**Thinking about communicating with health professionals during your maternity care…**

Q8: Did you feel **understood**?

- Always
- Mostly
- Sometimes
- Rarely
- Never
- *Prefer not to say*

Q9: Did you feel **listened to**?

- Always
- Mostly
- Sometimes
- Rarely
- Never
- *Prefer not to say*

Q10: Were things **explained to you in a way you could understand**?

- Always
- Mostly
- Sometimes
- Rarely
- Never
- *Prefer not to say*

Q11: Were you asked to **consent** prior to physical examinations in a way in which you felt that you could decline (say “no” to)?

*By physical examination, we mean touching your abdomen (‘tummy’ or ‘bump’), vagina (‘internal examination’), touching your breast during breastfeeding support and other examinations which involved touching your body*

- Always
- Mostly
- Sometimes
- Rarely
- Never
- *Prefer not to say*

Q12: Overall, did you feel **supported**?

- Always
- Mostly
- Sometimes
- Rarely
- Never
- Prefer not to say

Q13: Did you ‘mask’ when receiving care?

*By masking we mean things like not stimming, trying to ignore your sensory needs and communicating in a way that is more difficult for you to make things easier for the health professional*

- Always
- Mostly
- Sometimes
- Rarely
- Never
- Prefer not to say

**In the next sections of the survey, you will have space to tell us in your own words about your experiences at different time points.**

***We understand that pregnancy loss is a very upsetting subject for some people. Organisations who provide support for pregnancy loss are signposted at the end of the survey.***

Q14: Would you prefer to skip the next set of questions which are about pregnancy loss?

- Yes
- No

**[Yes skips to antenatal care section]**

## B. Pregnancy loss

**The questions in this section focus on pregnancy loss,** which includes pregnancies that ended without a live birth because of issues such as miscarriage, ectopic pregnancy and still birth**.**

Q1: Have you experienced a pregnancy loss?

*Please tick all that apply*

- Yes
- No
- Prefer not to say

[Skip: **No or Prefer not to say filters to antenatal section]**

Q2: If Yes, did you seek support from a health care professional?

- Yes on the one occasion, or every time I experienced pregnancy loss
- Yes on some of the occasions I experienced pregnancy loss
- No
- Prefer not to say

Q3: Please tell us about the factors that influenced your decision to contact a health care professional

[open text box]

**[No and Prefer not to say to Q2 filter to Q6 in this section after Q3]**

Q4: Did the health professionals treating you during pregnancy loss know that you were Autistic?

- Always
- Mostly
- Sometimes
- Rarely
- Never
- *Not applicable, I did Not know I was Autistic when I used maternity services*
- Prefer not to say

Q5. Did you try to communicate your needs around being Autistic whilst receiving health care for pregnancy loss?

*This could include a formal tool like an Autism Health Passport, some notes that you’ve written yourself, anything included in your maternity notes or speaking to staff*

- Always
- Mostly
- Sometimes
- Rarely
- Never
- *Not applicable, I did Not know I was Autistic when I used maternity services*
- Prefer not to say

Q6. If you would like to add anything about your experiences of pregnancy loss and healthcare for pregnancy loss, please do so here:

*You can write as much or as little as you like.*

*You could focus on your experiences of:*

- ***communicating*** *pain, concerns or needs, and health professionals’ response to them, including any misunderstandings*
- ***sensory*** *challenges*
- *receiving* ***information****, including if it met your needs*
- *How this made you feel*

[open text box]

Q7. Do you have any recommendations for how healthcare services for pregnancy loss could be made better for Autistic people?

[open text box]

Q8: Have you ever given birth?

*You can choose to include live births and any babies delivered following pregnancy loss if you would like the opportunity to tell us about the birth*

- Yes
- No, but I received antenatal care on at least one pregnancy
- No and I have not received any antenatal care

**[if answered ‘No and I have not received any antenatal care’ to skip to end of survey;**

**No but I received antenatal care on at least one pregnancy skips the birth section]**

## C. Antenatal care

**The questions in this section focus only on your antenatal care; there is another section that focuses on your birth experience later on.**

Q1: Did you receive antenatal care?

*Please tick all that apply*

- Yes I received NHS midwifery led care
- Yes I received NHS obstetric (doctor/consultant) led care
- Yes I received Private midwifery care
- Yes I receive Private obstetric care
- No I did Not receive any antenatal care
- Other
- Prefer not to say

[**only if answer ‘No I did not receive any antenatal care’ answerQ2; otherwise skip to Q3]**

Q2: If No, could you please tell us why you did not receive antenatal care?

[open text box]

**[after this question skip to birth questions]**

Q3: Did you have the same midwife or doctor each time for your antenatal care?

*Please choose one*

- Always
- Mostly
- Sometimes
- Rarely
- Never
- Prefer not to say

Q4: Did you choose any additional antenatal support?

*Please tick all that apply*

Private Doula

Private scan

Hypnobirthing

Parenting preparation classes (like NCT) face-to-face in person

Parenting preparation classes (like NCT) online

Online parenting preparation courses without interaction from other people (‘go at your own pace’)

Breastfeeding support groups whilst you were pregnant

Other (please specify) [open text box]

Q5: Did you experience any sensory issues during antenatal care?

*Please tick one option for each category*

**[matrix table – options: always to never 5 point scale]**

*Light (eg: bright light in examination room)*

*Sound*

*Smell*

*Touch during examination*

*Ultrasound gel on your tummy during examination*

*Other, please specify [open text box]*

Q6: Were antenatal health care staff aware that you were Autistic?

*By this we are asking if you had disclosed your autistic identity to maternity care professionals including midwives, sonographers and obstetricians.*

- Always
- Mostly
- Sometimes
- Rarely
- Never
- *Not applicable, I did Not know I was Autistic when I used maternity services*
- Prefer not to say

**[skip for: *Not applicable, I did Not know I was Autistic when I used maternity services* to Q65]**

Q7: If you told some staff and not others that you were Autistic, or things varied between pregnancies, please tell us why?

[open text box]

Q8: Did you try to communicate your needs around being Autistic whilst receiving antenatal health care?

*This could include a formal tool like an Autism Health Passport, some notes that you’ve written yourself or anything included in your maternity Notes or speaking to staff*

- Always
- Mostly
- Sometimes
- Rarely
- Never
- *Not applicable, I did not know I was Autistic when I used maternity services*
- Prefer not to say

Q9: If you have any additional comments regarding antenatal care, please use the box below

*You can write as much or as little as you like.*

*You could focus on your experiences of:*

- ***communicating*** *pain, concerns or needs, and health professionals’ response to them, including any misunderstandings*
- ***sensory*** *challenges*
- *receiving* ***information****, including if it met your needs*
- *How this made you feel*

[open text box]

Q10. Do you have any recommendations for how antenatal care could be made better for Autistic people?

[open text box]

## D. Birth

Have you ever given birth

Yes

No

**This section focuses on your birth experiences.**

Q1: In which years did you give birth? Please include each time you have given birth.

*For example: 2015, 2019, 2020*

[open text box]

Q2: Where did you give birth?

*Choose all that apply*

Midwifery led ward or birth centre

Obstetrician (also known as a doctor or consultant) led ward

At home

Other (please specify) [open text box]

Prefer not to say

Q3: Did you have one midwife or doctor care for you the whole way through your birth?

*By this we mean was there one individual per birth, not the same midwife for each of your births* if you have given birth more than once.

*Please choose one*

- Always
- Mostly
- Sometimes
- Rarely
- Never
- Prefer not to say
- Other (please specify) [open text box]

Q4: Did you experience any sensory issues during care for your birth?

*Please tick one option for each category*

**[matrix table – options: always to never 5 point scale]**

- *Light (eg: bright light in room)*
- *Sound*
- *Smell*
- *Touch during examination*
- *Ultrasound gel on your tummy during examination*
- *Other, please specify [open text box]*

Q5: Do you feel that your pain was manageable during birth?

*Please choose one*

- Always
- Mostly
- Sometimes
- Rarely
- Never
- Prefer not to say
- Other (please specify) [open text box]

Q6: Were health care staff at your birth or births aware that you were Autistic?

*By this we are asking if you had disclosed your autistic identity to maternity care professionals including midwives and obstetricians.*

- Always
- Mostly
- Sometimes
- Rarely
- Never
- *Not applicable, I did Not know I was Autistic when I used maternity services*
- Prefer not to say
- **[skip for: *Not applicable, I did Not know I was Autistic when I used maternity services* to Q65]**

Q7: If you told some staff and not others that you were Autistic, or things varied between births, please tell us why?

[open text box]

Q8: Did you try to communicate your needs around being Autistic whilst giving birth?

*This could include a formal tool like an Autism Health Passport, some Notes that you’ve written yourself or anything included in your maternity Notes or speaking to staff*

- Always
- Mostly
- Sometimes
- Rarely
- Never
- *Not applicable, I did Not know I was Autistic when I used maternity services*
- Prefer not to say

Q9: Did you prepare a birth plan?

- Yes for all births
- Yes for some births
- Unsure
- No
- Prefer not to say

Q10: Did your birth experience reflect your birth plan?

- Yes
- Partly
- No

**[if Yes, skip to Q12]**

Q11: If your birth experience was different to your birth plan, please explain how and why?

*You can write as much or as little as you like*

[open text box]

Q12: Please tell us anything you would like to about your experiences of receiving healthcare during birth

*You can tell us as much or as little as you would like*

*You could focus on your experiences of:*

- ***communicating*** *pain, concerns or needs, and health professionals’ response to them, including any misunderstandings*
- ***sensory*** *challenges*
- *receiving* ***information****, including if it met your needs*
- *How this made you feel*

[open text box]

Q13: Do you have any recommendations for how health care during birth could be made better for Autistic people?

[open text box]

## E. Infant feeding

Q1: What kind of milk did you feed your baby or babies?

*Please tick all that apply*

Breastmilk using your own milk (breastfeeding or expressing)

Breastmilk from a donor (‘donor milk’ or ‘human donor milk’)

Formula feeding

Not applicable

Prefer not to say

**[skips to relevant sections; not applicable and prefer not to say skip to the last section of the survey]**

### Breastfeeding questions

**This section is focused on your experience of breastfeeding.**

Q1: Did you find breastfeeding enjoyable or positive in some way?

- Always
- Mostly
- Sometimes
- Rarely
- Never
- *Prefer not to say*

Q2: How motivated were you to breastfeed your baby, even if you encountered difficulties?

- Always
- Mostly
- Sometimes
- Rarely
- Never
- *Prefer not to say*

Q3: How much research did you do to find out about breastfeeding?

- A lot
- Some
- A little
- None
- Prefer not to say

Q4: Did you experience pain when breastfeeding?

- Always
- Mostly
- Sometimes
- Rarely
- Never
- *Prefer not to say*

Q5: Did you find the intensity of breastfeeding difficult?

- Always
- Mostly
- Sometimes
- Rarely
- Never
- *Prefer not to say*

Q6: Did you find the unpredictability of your baby’s feeding patterns difficult?

- Always
- Mostly
- Sometimes
- Rarely
- Never
- *No, I fed baby on a schedule*
- *Prefer not to say*

Q7: Did you have sensory difficulties when breastfeeding?

*Sensory difficulties includes things like: being ‘touched out’ from the baby being close for so long and unpleasant sensations in the breast eg: from suckling or milk let down*

- Always
- Mostly
- Sometimes
- Rarely
- Never
- *Prefer not to say*

Q8: Did you express your breastmilk?

*Expressing uses your hand or a machine to take milk from the breast, which is often fed to the baby via a bottle, cup or syringe.*

- Always
- Mostly
- Sometimes
- Rarely
- Never
- *Prefer not to say*

Q9: Please tell us anything else about your experiences of breastfeeding or expressing your breastmilk.

*You may like to focus on challenges and anything that helped*

Q10: Did you receive support for breastfeeding from:

*Please tick all that apply*

Midwife

Health visitor

Lactation consultant (sometimes called an IBCLC)

Doula

Breastfeeding peer supporter in a one-to-one setting

Breastfeeding support group

Friends and family

Internet

Other, please specify [open text box]

I did not receive any breastfeeding support

**[skip if No support to next section]**

Q11: Please tell us about your experiences of receiving breastfeeding support

*You could include how much support, when and how useful it was*

[open text box]

Q12: Do you have any recommendations for how breastfeeding support could be made better for Autistic people?

[open text box]

### Formula feeding questions

**This section is focused on your experience of formula feeding.**

Q1: Which type of infant formula did you use?

*Tick all that apply*

- Ready-made liquid (in bottles)
- Powdered that you make up with hot water
- Prescription only formula

Q2: Did anybody give you support with learning how to formula feed your baby?

*Include choosing a type of milk and preparing bottles*

- *Yes*
- *No*
- *Can’t remember*
- *Prefer not to say*

Q3: Did you find it easy to select a type or brand of infant formula?

- Yes
- No
- Can’t remember
- Prefer not to say

Q4: Did you find it easy to understand the instructions on how to make up a bottle of formula?

- Yes
- No
- Can’t remember
- Prefer not to say

Q5: Did you find it enjoyable preparing bottles of formula?

- Always
- Mostly
- Sometimes
- Rarely
- Never
- *Prefer not to say*

Q6: Did you feel anxious about safely preparing formula?

- Always
- Mostly
- Sometimes
- Rarely
- Never
- *Prefer not to say*

*Q7: Did you find the unpredictability of baby’s feeding patterns difficult?*

- Always
- Mostly
- Sometimes
- Rarely
- Never
- *No, I fed baby on a schedule*
- *Prefer not to say*

Q8: Is there anything else you would like to tell us about your experiences of feeding your baby infant formula?

[open text box]

## F. Postnatal care

**This section is focused on the health care you experienced after birth.**

Q1: Did you or your baby need any inpatient care shortly after birth?

*Tick all that apply*

Maternity ward for me

Paediatric assessment unit for the baby

Neonatal unit for the baby

Other, please specify [open text box]

Q2: If Yes, was your birth companion allowed to stay with you/you and your baby?

- Always
- Mostly
- Sometimes
- Rarely
- Never
- I did Not have a birth companion

Q3: Did you experience any of these postnatal health issues?

*Tick all that apply*

*Mastitis (infection in the breast)*

*Infection in caesarean section scar*

*Postnatal depression*

*Postnatal anxiety*

*Postnatal psychosis*

*Incontinence*

*Prolapse (where the womb comes down into the vagina)*

Other, please specify [open text box]

Q4: Who did you receive post-natal health care from once you were discharged from hospital?

*Please tick all that apply*

Midwife

Health visitor

Doula

A nanny or maternity nurse

GP

Other, please specify [open text box]

Q5: What did you receive postnatal care for:

*Please tick all that apply*

Checking scars or stitches (from birth)

Pelvic floor (eg: for incontinence or prolapse)

Mental health (eg: for depression, anxiety or psychosis)

Breastfeeding

Other, please specify [open text box]

Q6: Please tell us anything else about your experiences of receiving postnatal healthcare

*You could choose to focus on feelings of being understood, supported and any impact of COVID*

[open text box]

Q7: Do you have any recommendations for how postnatal health care during could be made better for Autistic people?

[open text box]

Q8: *Did you receive support in the post natal period from…*

*Please tick all that apply*

*Partner*

*Family member*

*Friends*

*Doula*

*Support groups (face to face)*

*Support groups (online)*

Other, please specify [open text box]

Q9: Please tell us anything else you would like to add about your experiences of receiving support from family, friends and others (excluding health professionals) in the postnatal period

- [open text box]

**G. COVID and last thoughts**

This is the last section of the survey. There are five questions.

Q1: If you’ve been pregnant during the Covid-19 pandemic, and have any comments regarding how this affected your access to health care to do with pregnancy and maternity, please tell us about this below.

*You can write as much or as little as you like. You could focus on antenatal care, care when giving birth, and support postnatally*

[open text box]

Q2: If you have any last thoughts about anything to do with health care, Autism Health Passports or maternity care, please tell us here.

[open text box]

Q3: Would you like to:

- Be entered into the prize draw to win one of 10 £20 Amazon vouchers? yes/no
- Be emailed a copy of the study findings? Yes/No
- Be invited to take part in future studies about being Autistic and maternity care? Yes/No

**[if yes to any of these options, name/email boxes are shown]**

Name: [open text box]

Email Address: [open text box]

Q4: If you would like us to email you a copy of the findings, please leave your email address here if you did not give it already:

Email Address: [open text box]

**END**

This is the end of the questionnaire.

**[insert debrief here]**
