# Supplementary material for: “I am afraid of being treated badly if I show it”: A cross-sectional study of healthcare accessibility and Autism Health Passports among UK Autistic adults
Source: PLoS One. 2024 May 29;19(5):e0303873. doi: 10.1371/journal.pone.0303873 (PMC11135756; doi:10.1371/journal.pone.0303873)
Supplement: Attachment — Submitted filename: Response to reviewers v0a 6.3.24.docx [file pone.0303873.s002.docx]

Dear Dr Lund,

***Re: PONE-D-23-26665: “I am afraid of being treated badly if I show it”: a cross-sectional study of healthcare accessibility and Autism Health Passports among UK Autistic adults***

Thank you for your editorial notes and to the peer reviewer for their helpful comments. I have made the recommended changes to the manuscript highlighted in yellow on one copy, and also attach a clean copy. They are also described below alongside the reviewers’ feedback, with my response indented and italicised for clarity.

**Editor’s comments**

1. Please ensure that your manuscript meets PLOS ONE's style requirements, including those for file naming. The PLOS ONE style templates can be found at

<https://journals.plos.org/plosone/s/file?id=wjVg/PLOSOne_formatting_sample_main_body.pdf> and

[https://journals.plos.org/plosone/s/file?id=ba62/PLOSOne_formatting_sample_title_authors_affiliations.pdf](https://eur03.safelinks.protection.outlook.com/?url=https%3A%2F%2Fjournals.plos.org%2Fplosone%2Fs%2Ffile%3Fid%3Dba62%2FPLOSOne_formatting_sample_title_authors_affiliations.pdf&data=05%7C02%7CAimee.Grant%40Swansea.ac.uk%7C7452a34d49fd48dd71cc08dc3db4a95e%7Cbbcab52e9fbe43d6a2f39f66c43df268%7C0%7C0%7C638453093625756763%7CUnknown%7CTWFpbGZsb3d8eyJWIjoiMC4wLjAwMDAiLCJQIjoiV2luMzIiLCJBTiI6Ik1haWwiLCJXVCI6Mn0%3D%7C0%7C%7C%7C&sdata=qfy5HxhyQMC4J6Lo0b844hlmlI6tAENaEONLxH%2BMuVA%3D&reserved=0).

*These have been updated.*

2. Did you know that depositing data in a repository is associated with up to a 25% citation advantage (<https://doi.org/10.1371/journal.pone.0230416>)? If you’ve not already done so, consider depositing your raw data in a repository to ensure your work is read, appreciated and cited by the largest possible audience. You’ll also earn an Accessible Data icon on your published paper if you deposit your data in any participating repository ([https://plos.org/open-science/open-data/#accessible-data](https://eur03.safelinks.protection.outlook.com/?url=https%3A%2F%2Fplos.org%2Fopen-science%2Fopen-data%2F%23accessible-data&data=05%7C02%7CAimee.Grant%40Swansea.ac.uk%7C7452a34d49fd48dd71cc08dc3db4a95e%7Cbbcab52e9fbe43d6a2f39f66c43df268%7C0%7C0%7C638453093625761750%7CUnknown%7CTWFpbGZsb3d8eyJWIjoiMC4wLjAwMDAiLCJQIjoiV2luMzIiLCJBTiI6Ik1haWwiLCJXVCI6Mn0%3D%7C0%7C%7C%7C&sdata=4lTlxgIlYa3Q%2BgosZAfD7fV02IA03cfl5GdEQz%2B%2Bm0U%3D&reserved=0)).

*We did not request ethical approval for data sharing, so are unable to add our data to a repository.*

3. Thank you for stating the following in the Competing Interests section:

“SS is the research lead for Autistic Doctors International. KW is the research director for Autistic UK. The remaining authors declare no competing interests.”

We note that one or more of the authors are employed by a commercial company: Autistic UK & Autistic Doctors International

*Neither SS or KW receive any salary for their work with Autistic UK (a community interest company) or Autistic Doctors International (who do not legally exist as an organisation).*

*Additionally, I have taken on a non-executive director role for Disability Wales since the article was submitted for publication and have added it to the list of competing interests.*

A.        Please provide an amended Funding Statement declaring this commercial affiliation, as well as a statement regarding the Role of Funders in your study. If the funding organization did not play a role in the study design, data collection and analysis, decision to publish, or preparation of the manuscript and only provided financial support in the form of authors' salaries and/or research materials, please review your statements relating to the author contributions, and ensure you have specifically and accurately indicated the role(s) that these authors had in your study. You can update author roles in the Author Contributions section of the online submission form.

Please also include the following statement within your amended Funding Statement.

“The funder provided support in the form of salaries for authors [insert relevant initials], but did not have any additional role in the study design, data collection and analysis, decision to publish, or preparation of the manuscript. The specific roles of these authors are articulated in the ‘author contributions’ section.”

If your commercial affiliation did play a role in your study, please state and explain this role within your updated Funding Statement.

B.  Please also provide an updated Competing Interests Statement declaring this commercial affiliation along with any other relevant declarations relating to employment, consultancy, patents, products in development, or marketed products, etc.

Within your Competing Interests Statement, please confirm that this commercial affiliation does not alter your adherence to all PLOS ONE policies on sharing data and materials by including the following statement: "This does not alter our adherence to  PLOS ONE policies on sharing data and materials.” (as detailed online in our guide for authors <http://journals.plos.org/plosone/s/competing-interests>) . If this adherence statement is not accurate and  there are restrictions on sharing of data and/or materials, please state these. Please note that we cannot proceed with consideration of your article until this information has been declared.

Please include both an updated Funding Statement and Competing Interests Statement in your cover letter. We will change the online submission form on your behalf.

*In line with myself, SS and KW not receiving a salary, we have amended the competing interests statement to:*

*AG is a non-executive Director of Disability Wales. SS is the research lead for Autistic Doctors International, a peer support and advocacy group which is not a legal entity. KW is a non-executive director for Autistic UK a Community Interest Company.* This does not alter our adherence to PLOS ONE policies on sharing data and materials. *None of the three individuals receive a salary for these affiliations. The organisations did not have any role in the study design, data collection and analysis, decision to publish, or preparation of the manuscript. The remaining authors declare no competing interests.*

4. We note that you have indicated that there are restrictions to data sharing for this study. PLOS only allows data to be available upon request if there are legal or ethical restrictions on sharing data publicly. For more information on unacceptable data access restrictions, please see <http://journals.plos.org/plosone/s/data-availability#loc-unacceptable-data-access-restrictions>.

Before we proceed with your manuscript, please address the following prompts:

a) If there are ethical or legal restrictions on sharing a de-identified data set, please explain them in detail (e.g., data contain potentially identifying or sensitive patient information, data are owned by a third-party organization, etc.) and who has imposed them (e.g., a Research Ethics Committee or Institutional Review Board, etc.). Please also provide contact information for a data access committee, ethics committee, or other institutional body to which data requests may be sent.

b) If there are no restrictions, please upload the minimal anonymized data set necessary to replicate your study findings to a stable, public repository and provide us with the relevant URLs, DOIs, or accession numbers. For a list of recommended repositories, please see

<https://journals.plos.org/plosone/s/recommended-repositories>. You also have the option of uploading the data as Supporting Information files, but we would recommend depositing data directly to a data repository if possible.

We will update your Data Availability statement on your behalf to reflect the information you provide.

*I have updated the data availability statement at the beginning of the manuscript to provide greater clarity. It now states:*

“There are ethical restrictions on sharing a de-identified data set. The data contain sensitive and detailed information on healthcare experiences from a marginalized community. Accordingly, we did not seek ethical review, or gain participant consent, for data sharing.”

5. Please include captions for your Supporting Information files at the end of your manuscript, and update any in-text citations to match accordingly. Please see our Supporting Information guidelines for more information: <http://journals.plos.org/plosone/s/supporting-information>.

*This has been added to the manuscript below the references.*

**Additional Editor Comments (if provided):**

I agree with the reviewer that this manuscript has potential but could benefit from significant revision. In addition to addressing the points noted by the reviewer, please also introduce the nature and function of Autism Health Passports in the Introduction. This was vital context that was missing from the current version of the manuscript.

*Thank you for pointing out this omission, I have now added a sentence at the beginning of the third paragraph of the introduction to describe what Autism Health Passports are.*

**Reviewers' comments:**

**Reviewer #1:**

Thank you very much for the opportunity to review this interesting study. Your outcomes are important to the autistic community. Please see below some comments to improve the manuscript.

*Thank you very much for your supportive comments and careful attention when reviewing the paper.*

**Background:**

A generally well-written section.

Lines 65-66: I do not think that you can generalise this here. I agree that the experiences of autistic adults in healthcare need more reserach, but, I do not agree to cite a paper published 10 years ago (2014) and say that their experiences are not explored. For instance, you can find some great job done by medical imaging professionals around autistic people experiences in MRI, which I suggest citing (preferably in the discussion of your manuscript) to show the wider context of research aorud this topic.
<http://dx.doi.org/10.1089/aut.2022.0051>
<https://doi.org/10.1177/13623613211065542>
<https://doi.org/10.1186/s12913-023-10333-w>

*Thank you for pointing this out – you are correct that in the next paragraph we went on to describe the findings of many different studies into Autistic adults healthcare! We have updated the text to note recent growth in this area, including within MRI.*

Line 79: You state ''numerous documents'' but you only cite one. Please consider adding a couple of referneces here to support your statement.

*Apologies, we have added reference to another two policies and the Autism Act 2021.*

**Community involvement and reflexivity:**

I would suggest expanding a bit more regarding reflexivity. You can report gender, age, highest qualifications, ethnicity of the team, to allow the readers get a better idea of your views, experiences, and perceptions on the topic.

*We have added additional demographic details for each team member.*

**Survey design:**

I can see that you have not performed piloting with field experts, and also that you did not run any internal consistency tests (e.g. Cronbach's alpha). This means that you have to explicitly state in your limitations that you cannot quarantee either the validity or the reliability of the instrument.

*Thank you for highlighting this issue, we have added it in our limitations.*

**Participants and eligibility criteria:**

Line 129: I think you cannot state that multiple responses from the same participant were prevented by Qualtrics, since this survey software only prevents people to answer the survey twice from the same IP address. There is always the potentail risk of some people answering more than once from a different IP. Please change this sentence.

*Apologies, this sentence has been updated to reflect that it was IP addresses only that were limited.*

Lines 138-139: Did you inform the participants beforehand regarding the incentives given after the data collection? Was this written on the information sheet?
It seems that you collected some personal data to facilitate entry into the prize draw. Were the participants informed about this before entering the survey? This is important, since the anonymity of the respondents was not ensured. Was it a voluntary process, or were all the participants required to put their personal data in?

*Participants had the option to opt into the prize draw, but were not required to. This was made clear on the advert and participant information sheet. This information has been added into the text for clarity.*

**Analysis:**

Please consider providing the readers with the full terms for SPSS and ANOVA.

*These have been updated.*

I can see that you chose to analyse all data from respondents who had anwered at least their demographics. This is a correct strategy. However, was any attrition in your survey? Did you notice any respondents leaving the survey in different stages before completing it? If yes, you have to report attrition bias, and also to clarify if your quantitative analyses have taken the actual numbers of responses for each question, or the total number of responses, into account.

*Table 1 shows that the majority of the 193 (at least 191) completed these questions. Participants could leave questions blank if they did not wish to answer them. 93% of our participants answered at least 6 questions and were included in the ANOVA, and we already report this in the manuscript.*

Lines 156-157: You say here that you performed a thematic analysis. However, I cannot see any clear themes that derived from this in the results section. Thematic analysis is about identifying themes from the data. It seems that you performed a conceptual content analysis instead, since I can see reporting frequencies in the results of the open-ended questions. If this is the case, I suggest changing the above statement (and the ref as well), replacing this with content analysis, and adding a clarification about your strategy (e.g. how did you perform coding of data, how did you group them into common categories, etc.). In the results, you can always report the frequencies in which each category appears, and of course your representative quotes as you already did.

*We followed the Braun and Clarke thematic analysis process, although our data set mostly resulted in within-question codes being developed, with relatively fewer themes being developed. For this reason, we are happy to reenvisage our process through the lens of Schrier’s (2014) qualitative content analysis. We have described our process in more detail in the manuscript.*

**Results:**

Please consider changing your stategy regarding analysis and presentation of the qualitative data, as above.

*Done.*

Table 3: There is no need to put all these statistics into Table 3, since this turns out to be confusing to the reader. I suggest either depicting only the statistically significant results of the analyses, or taking the entire table out and leaving the text above which summarises well all significant results.

*We entirely agree with you, but the reporting of non-significant results is best practice under the APA publication guidelines. We would be happy to move this full table to a web appendix if the editor prefers.*

**Discussion:**

A well-balanced discussion summarising the key findings of the study. Perhaps consider only enriching this by adding some refs (like the ones suggested in the background) from different disciplines to show to highlight the problem in the light of the wider literature.

*We have added additional content to the discussion drawing on the wider	literature.*

**Limitations:**
This is fair enough, provided that you will add the limitations suggested above.

*This has been added.*
